# Large-area and bright pulsed electroluminescence in monolayer semiconductors

**DOI:** 10.1038/s41467-018-03218-8

**Published:** 2018-03-26

**Authors:** Der-Hsien Lien, Matin Amani, Sujay B. Desai, Geun Ho Ahn, Kevin Han, Jr-Hau He, Joel W. Ager, Ming C. Wu, Ali Javey

**Affiliations:** 1 Electrical Engineering and Computer Sciences, University of California, Berkeley, CA 94720 USA; 2 Materials Sciences Division, Lawrence Berkeley National Laboratory, Berkeley, CA 94720 USA; 3 Computer, Electrical and Mathematical Sciences and Engineering Division, King Abdullah University of Science & Technology, Thuwal, 23955-6900 Saudi Arabia

## Abstract

Transition-metal dichalcogenide monolayers have naturally terminated surfaces and can exhibit a near-unity photoluminescence quantum yield in the presence of suitable defect passivation. To date, steady-state monolayer light-emitting devices suffer from Schottky contacts or require complex heterostructures. We demonstrate a transient-mode electroluminescent device based on transition-metal dichalcogenide monolayers (MoS_2_, WS_2_, MoSe_2_, and WSe_2_) to overcome these problems. Electroluminescence from this dopant-free two-terminal device is obtained by applying an AC voltage between the gate and the semiconductor. Notably, the electroluminescence intensity is weakly dependent on the Schottky barrier height or polarity of the contact. We fabricate a monolayer seven-segment display and achieve the first transparent and bright millimeter-scale light-emitting monolayer semiconductor device.

## Abstract (web summary)

Atomically thin monolayers with high photoluminescence quantum yield are promising for optoelectronic and lighting applications. Here, the authors fabricate a transient-mode electroluminescent device to bypass the requirement of ohmic contacts for electrons and holes, and observe millimetre-scale light emission from a transparent 2D display.

## Introduction

Transition-metal dichalcogenides (TMDCs) such as WSe_2_ and MoS_2_ are semiconducting analogs of graphene, and are candidate materials for next-generation optoelectronic and electronic devices^1–5^. Their unique properties include naturally terminated surfaces at the monolayer limit (~0.7 nm), which when coupled with appropriate passivation of defect sites can result in near-unity photoluminescence (PL) quantum yield (QY)^3,6^. In addition, monolayer TMDCs display a myriad of attractive and unique physical properties including the lack of inversion symmetry, chiral light emission, and the ability to form heterostructures without the need for lattice matching^7–9^. Recent advances in the synthesis of high-quality TMDCs via chemical vapor deposition (CVD) demonstrate their potential for scalability^10,11^. The high PL QY and subnanometer thickness of TMDCs can be leveraged to develop large-area, transparent, and efficient light-emitting devices^3,6^. However, despite their exceptional material properties, a key challenge for TMDC light-emitting devices to date has been the formation of ohmic contacts to electrons and holes in the same device. Ohmic contacts in traditional light-emitting diodes (LEDs) are essential to minimize resistive losses and achieve high injection levels^12^. In previous works, steady-state electroluminescence (EL) was obtained in TMDCs using *p*–*n* junctions formed via electrostatic or chemical doping^13–15^. More recently, EL from complex quantum well heterostructures utilizing graphene with hexagonal boron-nitride tunnel barriers has been demonstrated^16,17^. However, the lack of suitable bipolar ohmic contacts remains to be a significant issue, ultimately limiting the performance of TMDC light-emitting devices^1^.

Inspired by the first EL device, the light-emitting-capacitor^18–21^, we achieve efficient bipolar carrier injection and light emission in TMDCs via transient-mode operation using a single metal-semiconductor contact (source). In this two-terminal device, the source is grounded and an AC voltage is applied to the gate electrode. Alternating electron and hole populations are injected into the monolayer TMDC from the source contact. Notably, the carrier injection is weakly dependent on the Schottky barrier height (*φ*_B_) (i.e., polarity of the contact) because of the large tunneling currents present at the source during the gate-voltage (*V*_g_) transients. The transient-EL (t-EL) device achieves bright EL at high injection levels. We demonstrate a millimeter-scale device with bright EL (peak power of 193 μW cm^−2^) from a ~0.7 nm thick monolayer in ambient room lighting. Finally, we show a large area device fabricated on a quartz substrate, which is transparent in the off-state by using indium tin oxide (ITO) electrodes.

## Results

### Operation and structure of the t-EL device

**Fig. 1:**
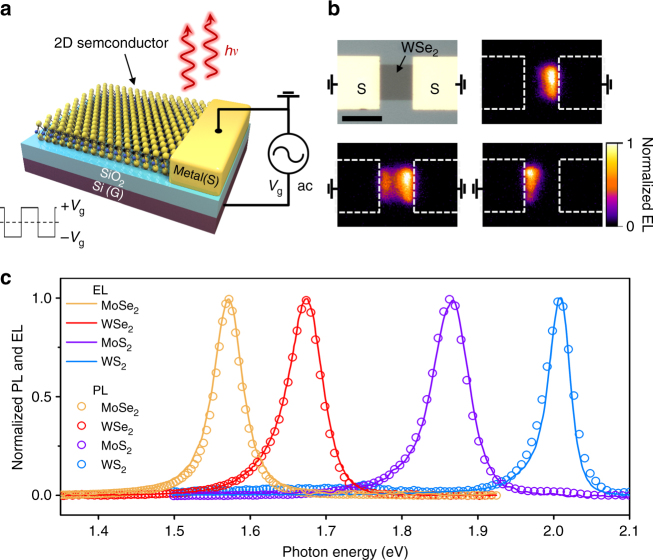
Transient EL in TMDCs. **a** Schematic of the t-EL device. An AC voltage is applied between the gate and source electrodes and emission occurs near the source contact edge. **b** Optical and EL image of a WSe_2_ device, showing that emission is only observed near the grounded source contacts. Scale bar is 10 μm. **c** EL and PL spectra measured for MoSe_2_, WSe_2_, MoS_2_, and WS_2_ monolayer devices

Figure 1a shows a schematic of the t-EL device, consisting of a monolayer TMDC on a heavily doped silicon substrate (gate) with a 50 nm thick SiO_2_ layer as the gate oxide. The TMDC is contacted with one metal electrode (source), and a bipolar square wave is applied between the gate and source. As shown in Fig. 1b, EL is only observed near the source contacts and the emission region laterally extends from the contact edge by ~3 μm (Supplementary Fig. 1). We fabricated devices based on four of the most heavily studied monolayer TMDCs by employing this generic device structure, specifically: WS_2_, MoS_2_, WSe_2_, and MoSe_2_^22,23^. All four of the studied materials show EL, with the spectral emission shape closely matching their respective PL (Fig. 1c).

### Carrier injection and light-emission mechanism

**Fig. 2:**
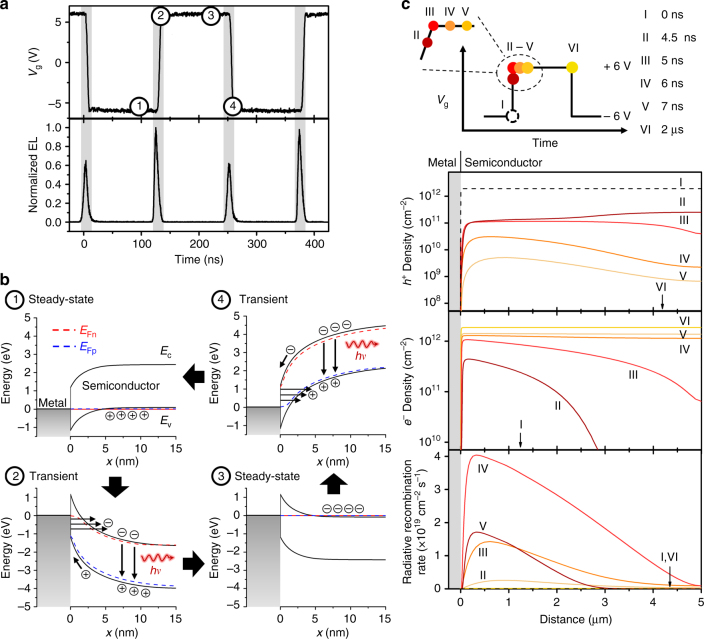
Operation mechanism. **a** Time-resolved electroluminescence and the corresponding *V*_g_, showing that EL occurs at the *V*_g_ transients (time points 2 and 4). **b** Band diagrams at different times during the operation cycle, corresponding to **a**. *E*_Fn_ and *E*_Fp_ indicate the quasi-Fermi levels for electrons and holes, respectively. **c** *V*_g_ pulse applied to the simulated device and the corresponding electron/hole density and radiative recombination rate. Simulations were performed for material parameters corresponding to WSe_2_ using a 50 nm thick gate oxide and *V*_g_ = ± 6 V (simulated band diagrams are also shown in Supplementary Figs. 5 and 6)

We performed time-resolved EL (TREL) measurements to understand the dynamic performance and the mechanism of light emission in the t-EL device. The measured TREL from a WSe_2_ device and the corresponding *V*_g_ square wave are shown in Fig. 2a and Supplementary Fig. 2 (the operation mechanism is also depicted in Supplementary Video 1). Pulsed EL is observed at each *V*_g_ transition and has a full-width half-maximum of 8 ns. EL emission in the device increases linearly with frequency (*f*) as shown in Supplementary Fig. 3 and Supplementary Note 2, with no changes in spectral shape. Note that the EL is stable with an  approximately ±25%) intensity variation over time (Supplementary Fig. 4). The emission mechanism can be elucidated from the sequence of energy band diagrams (simulated via Sentaurus TCAD) shown in Fig. 2b, Supplementary Figs. 5 and 6 as well as the carrier densities and radiative recombination rate shown in Fig. 2c. When *V*_g_ is held at −6 V, the hole density in the semiconductor is large and approaches its steady-state value (*p*_0_~1.9 × 10^12^ cm^−2^). When *V*_g_ is switched to +6 V, the field across the capacitive component of the device (i.e., SiO_2_ gate dielectric) cannot change instantaneously. As a result, the applied voltage is dropped across the resistive parts of the device including the semiconductor and the source contact, but is dominated by the latter. The large voltage drop and the steep energy band bending at the Schottky contact lead to large transient tunneling currents. Injected electrons diffuse inward while holes exit the semiconductor through the contact or recombine with incoming electrons. Thus, the hole density shows a continuous decrease, whereas the electron density in the semiconductor increases until it reaches its steady-state value (*n*_0_~1.9 × 10^12^ cm^−2^). At steady state, the band bending in the semiconductor and at the contact decreases (Supplementary Figs. 5 and 6) and the tunneling currents subside. The excess electron and hole populations simultaneously present (large quasi-Fermi level splitting) during the AC transient result in pulsed light emission. Similarly, this mechanism can also explain the emission from the device during a −6 V to +6 V *V*_g_ transient. The large transient tunneling currents in the t-EL device allow for efficient modulation of the carrier densities in the semiconductor, surmounting the large Schottky barriers typically associated with non-ohmic contacts to TMDCs. Simulated transient currents are shown in Supplementary Fig. 7 and the various current components present are discussed in detail in the Supplementary Note 3.

### Schottky barrier height and gate-voltage dependence

**Fig. 3:**
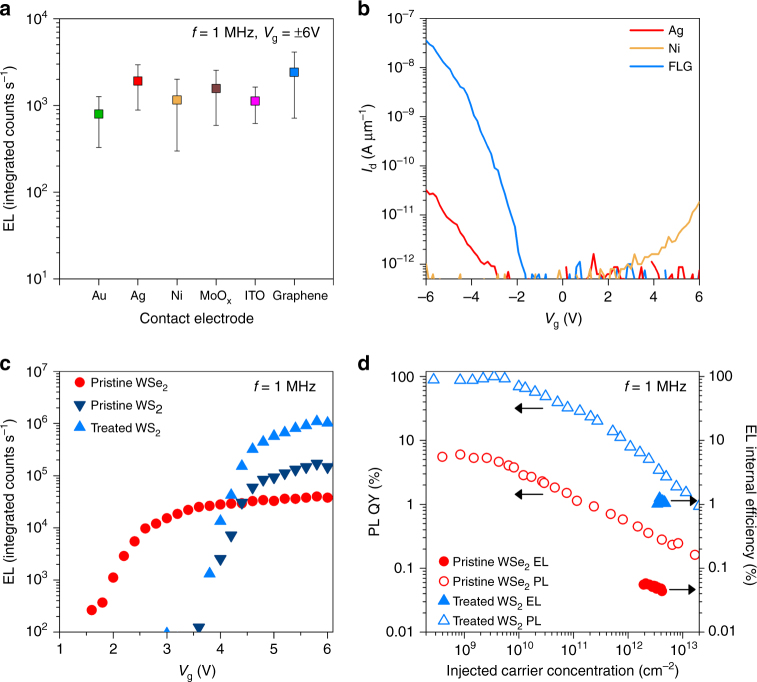
Contact and voltage dependence. **a** EL from WSe_2_ devices fabricated using various source contacts. Error bars indicate standard deviation of EL intensity measured from five or more different devices. **b** *I*_d_–*V*_g_ characteristics of WSe_2_ devices contacted by Ag, Ni, and few-layer graphene source electrodes. **c** Voltage dependence of EL for WSe_2_ and WS_2_ devices (WS_2_ before and after superacid treatment). **d** PL QY and EL internal efficiency measured for a WSe_2_ device and a superacid-treated WS_2_ device as a function of injected carrier concentration

The impact of *φ*_B_ on transient carrier injection and light emission is further studied by fabricating WSe_2_ devices with electrodes prepared by sputtering (ITO), thermal evaporation (Au, Ag, Ni, MoO_x_), as well as transferred van der Waals few-layer graphene contacts. The different contacts result in ~3 orders of magnitude variation in the on-current when the device is configured as a transistor (Supplementary Fig. 8). However, the corresponding t-EL devices show a maximum variation in the integrated emission intensity of only ~4× as shown in Fig. 3a (error bars indicate standard deviation of EL intensity measured from five or more different devices). WSe_2_ transistor characteristics for Ag, Ni, and graphene contracts are shown in Fig. 3b. Sentaurus TCAD simulations similarly show negligible difference in integrated EL for varying *φ*_B_ over the range of ohmic (*φ*_B_ = 0.05 eV) to mid-gap (*φ*_B_ = *E*_g_/2) (Supplementary Fig. 9). The relative intensity of emission during the two *V*_g_ transients, however, does vary for devices with varying *φ*_B_. As shown in the TREL of a hole selective contact device (Supplementary Fig. 10), the EL intensity at +*V*_g_ to −*V*_g_ transient is stronger than the other transient. This is consistent with simulation results (Supplementary Fig. 11) and discussed in the Supplementary Note 4. In addition to the effect of *φ*_B_, we also studied the dependence of EL on varying *V*_g_ (Fig. 3c and Supplementary Fig. 12). EL is observed from the device when *V*_g_ is greater than the turn-on voltage (*V*_t_), whose precise value is dependent on the bandgap (*E*_g_) of the material and parasitic resistances in the device. We experimentally observe a higher *V*_t_ for WS_2_ (4.1 V) as compared to WSe_2_ (2.0 V) which is qualitatively consistent with the larger *E*_g_ of WS_2_^24^.

The efficiency in traditional light-emitting devices is defined as the number of emitted photons over the total current. However, for the t-EL device, transient current measurement is challenging due to device and measurement setup parasitic capacitances as well as the high slew rate (1.6 V ns^−1^) of the *V*_g_ square wave. Given this limitation, we define EL internal efficiency (*η*_i_) based on the total steady-state carrier concentration, which represents the maximum number of carriers that can undergo radiative recombination in a given *V*_g_ cycle. The efficiency is thus defined as: $\eta _i = \frac{{{\mathrm{photons}}/{\mathrm{cycle}}}}{{(n_{\it{0}} + p_{\it{0}}) \times {\mathrm{area}}}}$ (Supplementary Equation 6). The sum of the steady-state concentrations, (*n*_0_ + *p*_0_) is given by *C*_g_(2*V*_g_-*E*_g_ *q*^−1^*)q*^−1^ (Supplementary Equation 7). Here, *n*_0_ and *p*_0_ are the steady-state electron and hole concentrations corresponding to a positive and negative *V*_g_, respectively, *C*_g_ (69.1 nF cm^−2^ for 50 nm SiO_2_ gate oxide) is the areal gate capacitance and *q* is the elementary charge. The analytical value of *n*_0_ + *p*_0_ closely matches that from simulations at sufficiently high *V*_g_ (Fig. 2c and Supplementary Fig. 13). *η*_i_ approaches 100% for the case where the PL QY is unity and all the steady-state carriers present in the device undergo recombination during a *V*_g_ transient. In practice, the PL QY may not be 100%, and only a fraction of the steady-state carriers will undergo recombination in the semiconductor, while the remainder exit through the contact due to the finite slew rate of the AC source. Further details on the efficiency calculation are provided in the Supplementary Note 6.

The WSe_2_ t-EL device at *n*_0_ + *p*_0_ = 2.1 × 10^12^ cm^−2^ (calculated from Supplementary Equation 7 for *V*_g_ = 3.2 V and *E*_g_ = 2.34 eV) has an EL external efficiency (*η*_e_) of 0.01% (Supplementary Fig. 14)^24^. The *η*_i_ of this device is extracted from *η*_e_ by considering the escape cone and optical interference from the Si substrate with a 50 nm SiO_2_ layer^3,25^. Using this method, we calculate *η*_i_ of ~0.06%. To contrast the performance of the t-EL device relative to the PL QY of the material, the steady-state and quasi-steady-state PL are measured using a continuous-wave and pulsed laser respectively (Fig. 3d, Supplementary Note 1, and Supplementary Fig. 15). The droop observed in PL QY at high injection levels has been previously attributed to biexcitonic recombination in two-dimensional (2D) semiconductors^3,6^. The calculated *η*_i_ for WSe_2_~0.06% has an upper bound equal to the PL QY of the material (~0.3%) (Fig. 3d). The efficiency of the device can be improved by utilizing a material with a high PL QY. We fabricated WS_2_ devices where the semiconductor surface is passivated using a non-oxidizing organic superacid: bis(trifluoromethane)sulfonimide, which has been shown to enhance the PL QY at low injection levels (<10^9^ cm^−2^) to >95%^3,6^. In this superacid-treated device, we obtain a peak *η*_e_ of ~0.27% at *n*_0_ + *p*_0_~3.8 × 10^12^ cm^−2^ (calculated from Supplementary Equation 7 for *V*_g_ = 5.8 V and *E*_g_ = 2.88 eV), corresponding to *η*_i_~1.2% (the EL for this device before and after treatment are shown in Supplementary Fig. 16)^24^. We note that the *η*_i_ is still significantly limited by the PL QY droop at high injection levels (3.4%) (Fig. 3c and Supplementary Fig. 17). The effect of biexcitonic recombination on the PL QY is shown in Supplementary Fig. 18. This indicates that for pump levels in the range of 10^12^ cm^−2^, WS_2_ that has a *C*_bx_ of ~0.1 cm^2^ s^−1^, *η*_e_ can be improved to ~40% by sufficiently lowering *C*_bx_. The next phase for improving the efficiency involves engineering the radiative lifetime and biexcitonic recombination rate by properly selecting the substrate and/or top coating. Similar efforts are made in III–V thin film devices, where the radiative recombination rate is highly dependent on the optical mode density and refractive index of the medium^26^.

### Seven-segment display and millimeter-scale t-EL device

**Fig. 4:**
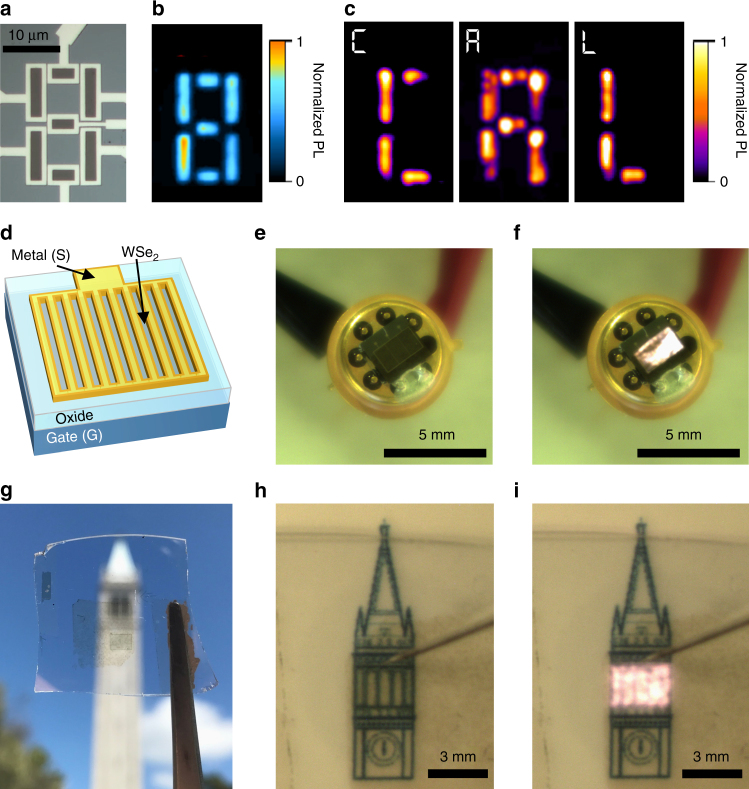
Seven-segment display and millimeter-scale t-EL device. **a** Optical microscope image and **b** photoluminescence image of a seven-segment t-EL display. **c** Operation of the seven-segment display showing EL in the shape of C–A–L. **d** Schematic of a millimeter-scale device, showing the grid source electrode structure to increase active emission area. **e**, **f** Photograph of a packaged, 3 mm × 2 mm, device in the off **e** and on **f** state. **g** Photograph of a millimeter-scale transparent device. **h**, **i** Photograph of a large area (3 mm × 2 mm) transparent device in the off **h** and on **i** state

To demonstrate the versatility of this transient injection mechanism, we fabricated the first display using TMDCs as the light-emitting material. A seven-segment display was fabricated using monolayer WSe_2_ with Ni contacts. An optical image and the corresponding PL image of the display are shown in Fig. 4a, b, respectively. By grounding the source electrodes of individual elements in the display sequentially, we show that we can dynamically display the letters C–A–L. Furthermore, we show that the t-EL device can be readily scaled to millimeter dimensions by using large-area monolayer films of WSe_2_ grown by CVD^11^. In order to maximize the light-emitting area, a Ni electrode is patterned in a grid structure with a line spacing of 8 μm (corresponding to ~2× of the emission FWHM, Supplementary Fig. 1). As a result, the EL from each contact edge fills the active area as shown schematically in Fig. 4d. The final device was then packaged in a standard chip carrier. Figure 4e, f show photographs of a 3 mm × 2 mm device in operation under room lights. The average and peak output power of the device operating at *f* = 400 kHz, was measured directly using a power meter to be 0.62 μW cm^−2^ and 193 μW cm^−2^, respectively. This shows that a monolayer semiconductor has the potential to be used in display and lighting applications. EL photographs of another device as well as the EL spectrum of the mm-scale device are shown in Supplementary Fig. 19. Finally, we demonstrate that the device can be made transparent as shown in Fig. 4g, with the corresponding transmission spectrum of the device shown in Supplementary Fig. 20. This device is fabricated on a fused quartz substrate, utilizing ITO as the gate and source electrodes and Al_2_O_3_ as the gate dielectric. Photographs of a transparent device in the off- and on-state are shown in Fig. 4h, i, respectively. Videos showing operation of both the transparent and opaque millimeter-scale devices are available in the Supplementary Information (Supplementary Videos 2 and 3, respectively).

In summary, we have demonstrated bright EL at high injection levels using transient-mode operation via a simple dopant-free device that does not require complex heterostructures to achieve light emission. EL from this device is weakly dependent on the Schottky barrier height or polarity. The transient-EL concept demonstrated in this work can be extended to large bandgap materials in the future, for which achieving ohmic contacts to both carrier types is particularly challenging. We show that this device structure can be scaled-up to obtain light emission on millimeter length scales. The ability to realize large-scale light emission from monolayer semiconductors opens numerous potential opportunities in the field of 2D materials and can lead to the realization of transparent displays. Our results also suggest that the main factors limiting the performance of the t-EL device is PL QY droop due to biexcitonic recombination. The device performance can be further improved through engineering of the optical medium^3,25^. Finally, unlike traditional LEDs, the exposed light-emitting surface of these devices also permits for the direct integration of plasmonic structures, nano-antennas, and photonic crystals, which could allow for the fabrication of high-speed devices or the development of electrically pumped 2D lasers^27–29^.

## Methods

### Device fabrication

Devices based on MoS_2_ (SPI supplies), WS_2_ (HQ graphene), and MoSe_2_ (HQ graphene) were fabricated from monolayers mechanically exfoliated onto 50 nm SiO_2_/Si p^++^ substrates. The WSe_2_ monolayers used in this work were synthesized by CVD with conditions tuned to optimize the PL QY of the material. Growth methods are presented in the reference^11^, with the following modifications to obtain large area films: the Se boat temperature was ramped up to 130 °C and the growth time was extended to 42 min. The typical PL QY of synthesized WSe_2_ samples at low pump powers is in the range of 5–15%. CVD WSe_2_ monolayers were subsequently transferred to the desired substrate. For large area devices, WSe_2_ films were transferred using a technique reported in reference^30^ either to Si/SiO_2_ or fused quartz/ITO/Al_2_O_3_ substrates. For patterning micron-scale devices on Si/SiO_2_, the 2D material was first etched using XeF_2_, while micron-scale devices on fused quartz/ITO/Al_2_O_3_ were etched using O_2_/CF_4_ plasma. Metal electrodes were subsequently deposited by thermal evaporation or sputtering. For graphene contacted devices, graphene flakes (three to five layers thick) were etched and subsequently placed on the TMDC using a previously reported dry transfer technique^9^. Due to the low PL QY of as-exfoliated MoS_2_, devices were prepared using 40 nm thick Au contacts and subsequently were chemically treated using an organic superacid^3^ to enhance the PL QY prior to EL measurements. All patterning was performed using electron-beam lithography with either poly (methyl methacrylate) (PMMA) or methyl methacrylate/PMMA as the resist. In the case of devices fabricated on quartz substrates, a 10 nm Au film was thermally evaporated on the PMMA and etched post e-beam lithography using KI/I_2_ etchant. For the case of superacid-treated WS_2_ devices shown in this work, few-layer graphene contacts were used. This was done in order to eliminate process-induced degradation of the 2D layer, which predominantly occurs during e-beam lithography.

### Electrical and optical characterization

Electroluminescent devices were pumped using a bipolar square wave from an Agilent 33522A arbitrary waveform generator applied to the gate electrode, while the source contact was grounded. The PL and EL data presented in this work was measured using a custom built micro-PL instrument described in detail in reference^3^ (Supplementary Methods). To measure PL, either a 514.5 nm Ar^+^ laser or a monochromated 514 nm line from a pulsed supercontinuum laser was used as the excitation source. PL and EL were collected using either a 50× ultra-long working distance or a ×10 objective lens. The PL signal was passed through a 550 nm dielectric longpass filter to block the excitation light. Both PL and EL were dispersed by a spectrometer with a 340 mm focal length and 150 groove per mm grating, and detected using a Si charge-coupled device (CCD) (Andor iDus BEX2-DD). Prior to each measurement, the CCD background was obtained and subsequently subtracted from the PL/EL acquisition. Time-resolved EL measurements were collected using a time-correlated single-photon counting (TCSPC) module. The signal was detected with a low dark count avalanche photodiode operating in single-photon counting mode (IDQuantique). The timing for the TCSPC was determined using pulses generated with the same phase/frequency as the square wave applied to the gate of the device. PL and EL imaging were performed in a fluorescence microscopy setup using a 470 nm LED as the excitation source and a CCD detector (Andor Luca) to acquire images. For EL imaging, the illumination source was turned off. Macroscopic photographs and videos of EL were taken using a CMOS camera with a telephoto lens; images are single exposures taken in ambient room lighting. Note that for the macroscopic EL photographs, the cold filter was removed from the camera. All measurements reported in this paper are taken at room temperature, in an ambient lab environment, with no device encapsulation. Transistor *I*_d_–*V*_g_ characteristics as well as *C*–*f* measurements of the gate oxide (Supplementary Fig. S21 and Supplementary Note 5) were taken using an Agilent B1500A semiconductor parameter analyzer.

### Device simulations

Simulations were performed in Sentaurus TCAD, where the device structure consists of a monolayer of WSe_2_, with the following material parameters used: *E*_g_ = 2.34 eV, *ε* = 4*ε*_0_, *µ*_*n*_ = *µ*_*p*_ = 100 cm^2^ V^−1^ per s, *τ*_*n*,SRH_ = *τ*_*p*,SRH_ = 2.5 ns, *m*_e_* = *m*_h_* = 0.345 *m*_0_, and a SiO_2_ gate oxide thickness of 50 nm. A non-local tunneling model accounts for the dependence of tunneling currents at the metal-semiconductor contact. Simulation snapshots record the transient device characteristics, recombination rates, and carrier densities, at several different instances, immediately before, during and after a *V*_g_ pulse edge. The slew rate of the gate-voltage pulse used in the transient simulations is 2.4 V per ns. For simulations shown in Fig. 2, Supplementary Figs. 5, 6, and 13 ambipolar contacts (*φ*_Bn_ = *φ*_Bp_ = *E*_g_/2) were used. The recombination models employed here are based on free carriers and material properties for GaAs were used for all parameters not specified above; however, the simulation adequately captures the physics of device operation and light emission qualitatively. For accurate quantitative analysis of this device, first-principle calculations and inclusion of the exciton physics of 2D materials are needed.

### Data availability

The data that support the findings of this study are available from the corresponding author upon request.

## Electronic supplementary material

Supplementary Information

Description for the Supplementary Movies

Supplementary Movie 1

Supplementary Movie 2

Supplementary Movie 3
