# Supplementary material for: Large-area and bright pulsed electroluminescence in monolayer semiconductors
Source: Nat Commun. 2018 Mar 26;9:1229. doi: 10.1038/s41467-018-03218-8 (PMC5955902; doi:10.1038/s41467-018-03218-8)
Supplement: Supplementary file 1 — Supplementary Information [file 41467_2018_3218_MOESM1_ESM.pdf]

Supplementary Figures

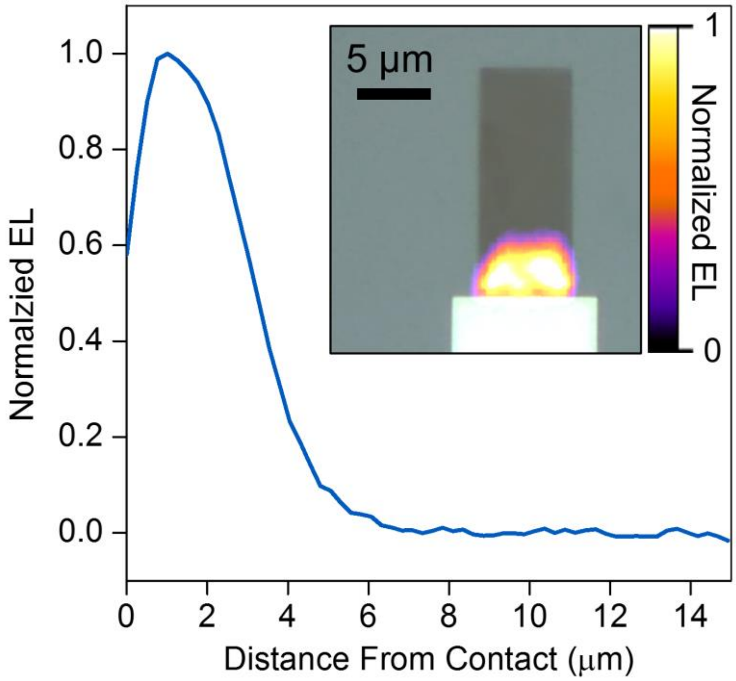

Supplementary Figure 1 | Spatial profile of EL. Spatial profile of EL intensity for a WSe_2 t-EL device fabricated with a single source contact. Inset shows superimposed optical and EL image. Measurement was taken with $V_g = \pm$ 9V at a frequency of 1 MHz. The EL peak is ~1 μm from the contact edge with a FWHM of ~3.3 μm; note that the experimental values are limited by the resolution of the optical system (~500 nm).

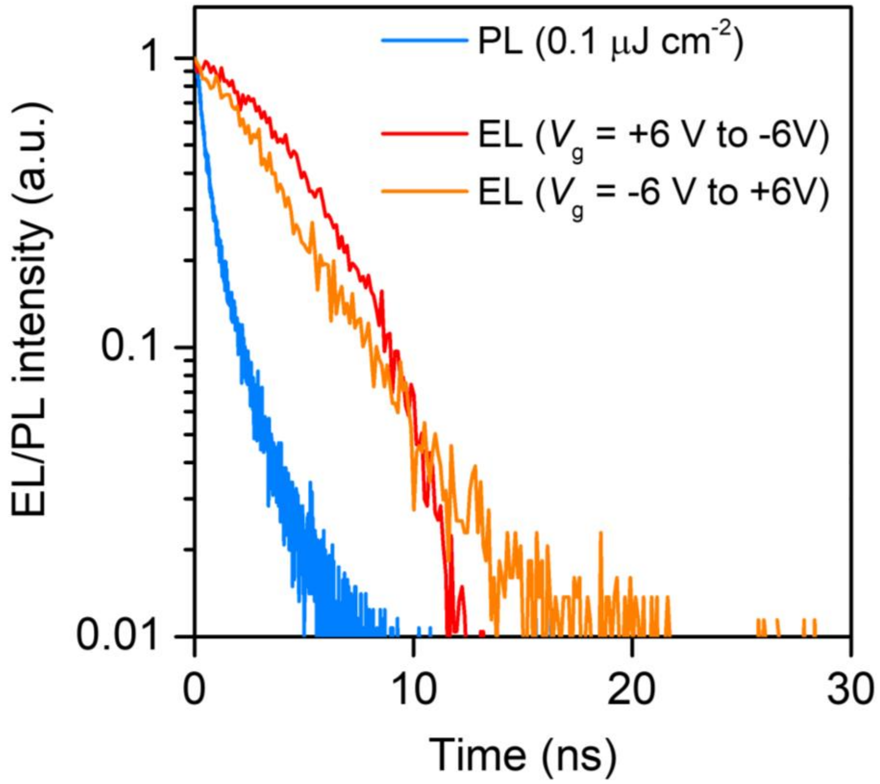

Supplementary Figure 2 | TRPL and TREL decay. Time resolved PL decay measured at an injection level of 0.1 μJ cm^{-2} and time resolved EL decay measured at gate transitions of V_g = +6 V to -6 V and V_g = -6 V to +6 V.

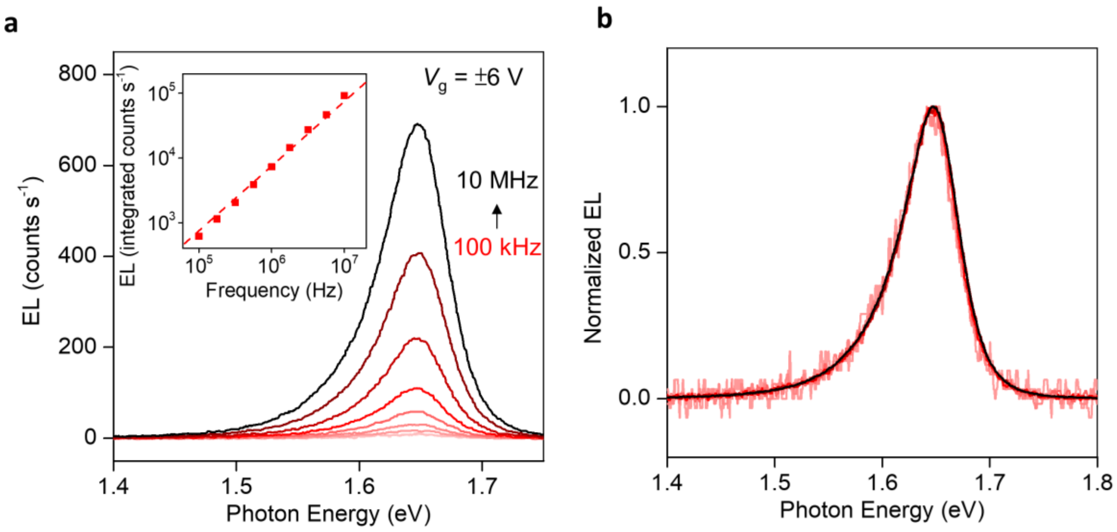

Supplementary Figure 3 | Frequency dependence. (a) EL spectra of a WSe_2 device operated at gate frequencies ranging from 100 kHz to 10 MHz. Inset shows integrated EL counts per cycle as a function of operating frequency. The superlinar increases EL versus frequency is attributed to time dependent variation in the device (Supplementary Fig. 4). **(b)** Normalized EL spectra of Supplementary Fig. 3a.

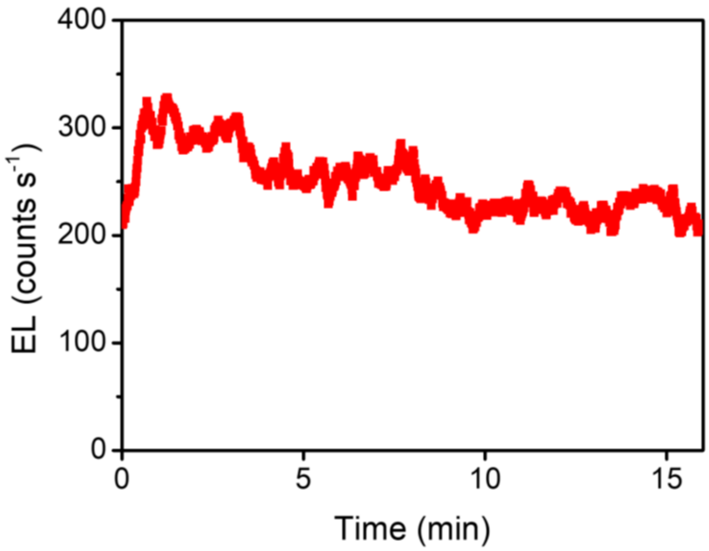

Supplementary Figure 4 | Device stability. EL counts measured for a WSe_2 device operated for fifteen minutes in ambient with no encapsulation. The device shows a maximum variation of 50% over the full operation time.

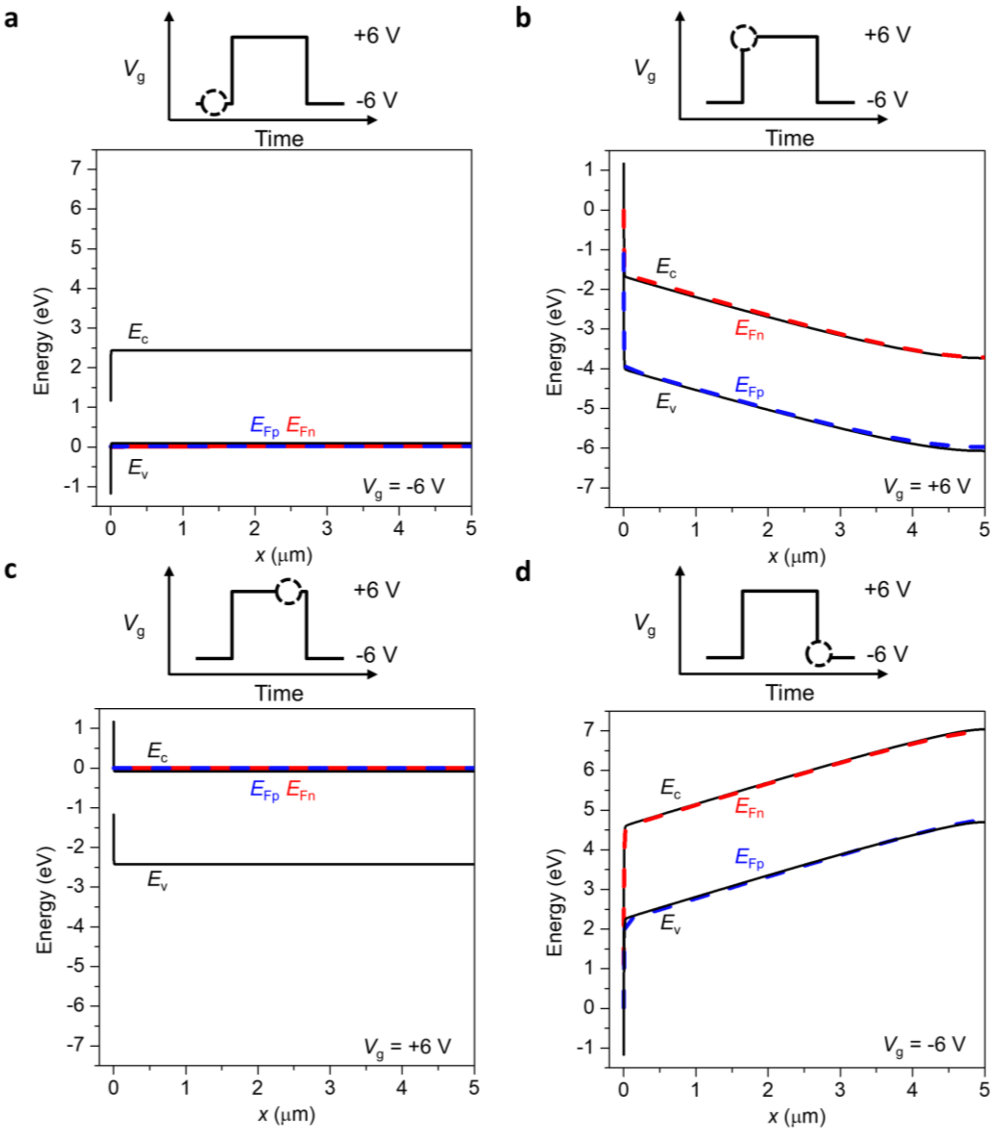

Supplementary Figure 5 | Simulated band diagrams. (a) Band diagram at V_g = -6 V, immediately before V_g transient. **(b)** Band diagram immediately after switching bias to V_g = +6 V. **(c)** Band diagram at V_g = +6 V, immediately before V_g transient. **(d)** Band diagram immediately after switching bias to V_g = -6 V. Simulations were performed for material parameters corresponding to WSe_2, further details are provided in the methods.

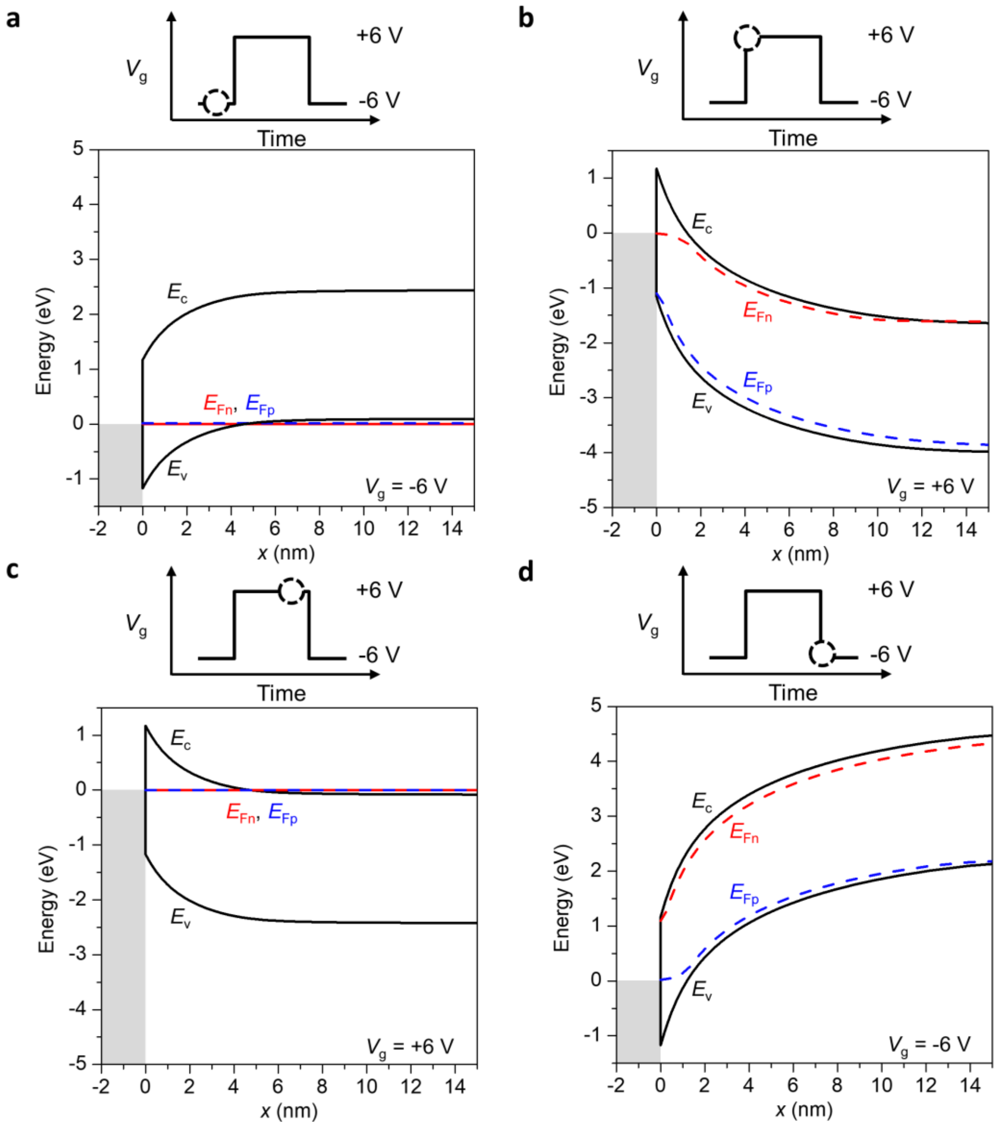

Supplementary Figure 6 | Simulated band diagrams near the contact edge. Data shown here is from Supplementary Fig. 5, expanded near the Schottky contact for clarity. **(a)** Band diagram at V_g = -6 V, immediately before V_g transient. **(b)** Band diagram immediately after switching bias to V_g = +6 V. **(c)** Band diagram at V_g = +6 V, immediately before V_g transient. **(d)** Band diagram immediately after switching bias to V_g = -6 V. Simulations were performed for material parameters corresponding to WSe_2, further details are provided in the methods.

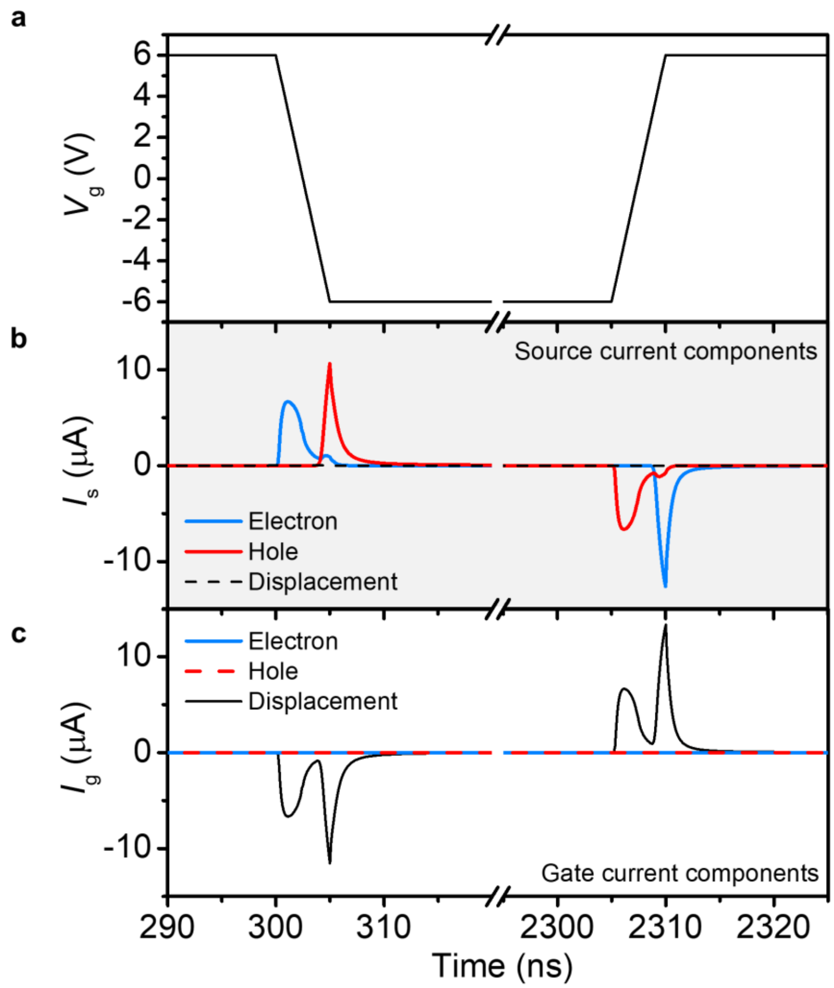

Supplementary Figure 7 | Transient current components. (a) V_g as a function of time. Corresponding source (I_s) and gate (I_g) currents are shown in **(b)** and **(c)**, respectively. I_s is dominated by electron and hole components, while the displacement current is relatively negligible. I_g is dominated by the displacement current. Simulations were performed for material parameters corresponding to WSe_2, further details are provided in the methods.

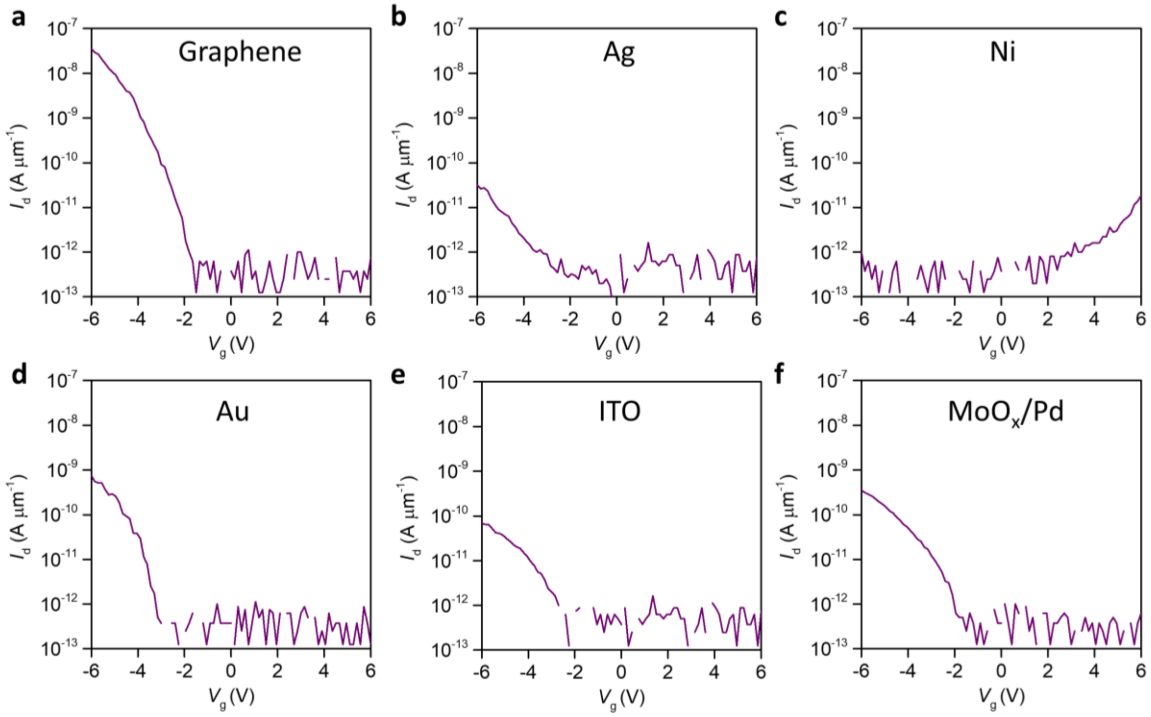

Supplementary Figure 8 | I_d-V_g characteristics of WSe_2 devices with various contacts. I_d-V_g characteristics of representative WSe_2 devices shown in Fig. 3a, fabricated with **(a)** transferred few-layer graphene (FLG) contacts, **(b)** thermally evaporated Ag, **(c)** thermally evaporated Ni, **(d)** thermally evaporated Au **(e)** sputtered ITO, and **(f)** thermally evaporated MoO_x/Pd[1]. $|V_{ds}| = 1$ V for all cases.

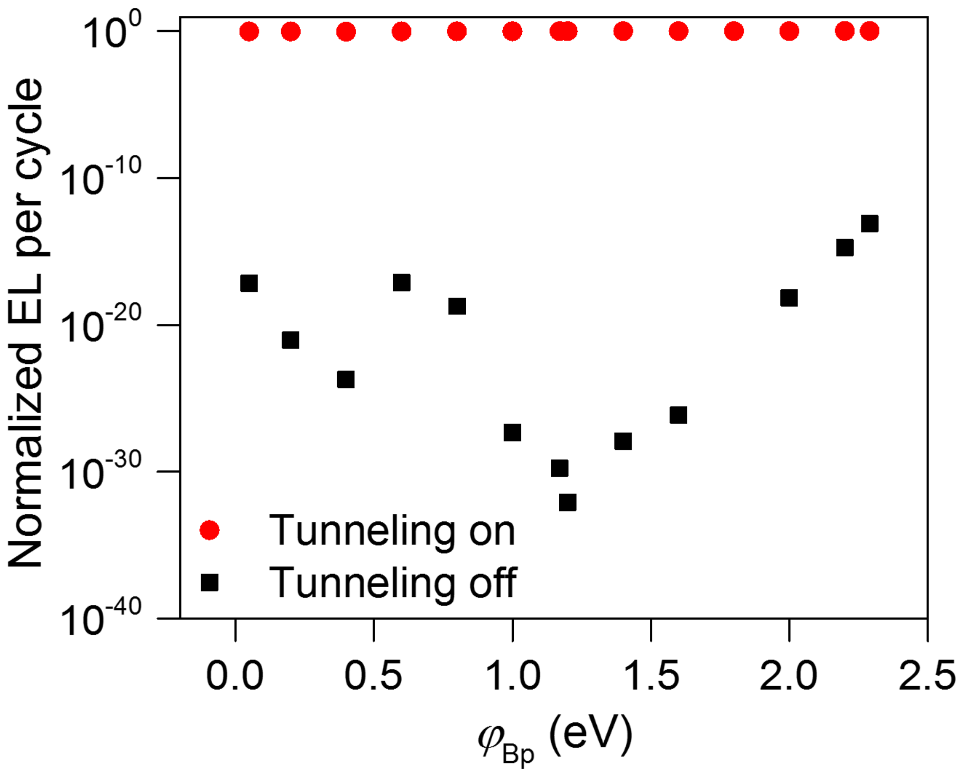

Supplementary Figure 9 | Dependence of EL on Schottky barrier height. Simulated EL integrated per gate cycle for a device where the Schottky barrier height is varied (note that φ_{Bn} + φ_{Bp} = E_g). Simulations were performed in the case where tunneling was allowed (red circles) and unaccounted for (black squares). Simulated EL shows negligible dependence on the Schottky barrier height when we account for the tunneling current. The integrated EL intensity for the case where the tunneling model is off is orders of magnitude lower and is dependent on the Schottky barrier height and several simulation parameters such as gate work function, which was assumed to be 5.1 eV. Simulations were performed for material parameters corresponding to WSe_2, further details are provided in the methods.

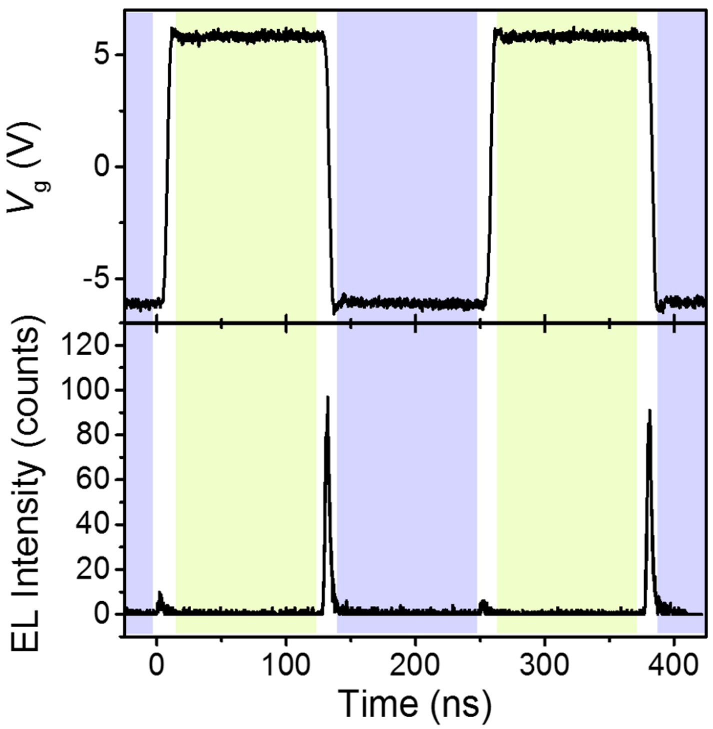

Supplementary Figure 10 | TREL of a WSe_2 device with unipolar MoO_x/Pd contacts. TREL measured for a WSe_2 device with unipolar MoO_x/Pd contacts, which show a large Schottky barrier to electrons and a low Schottky barrier to holes.

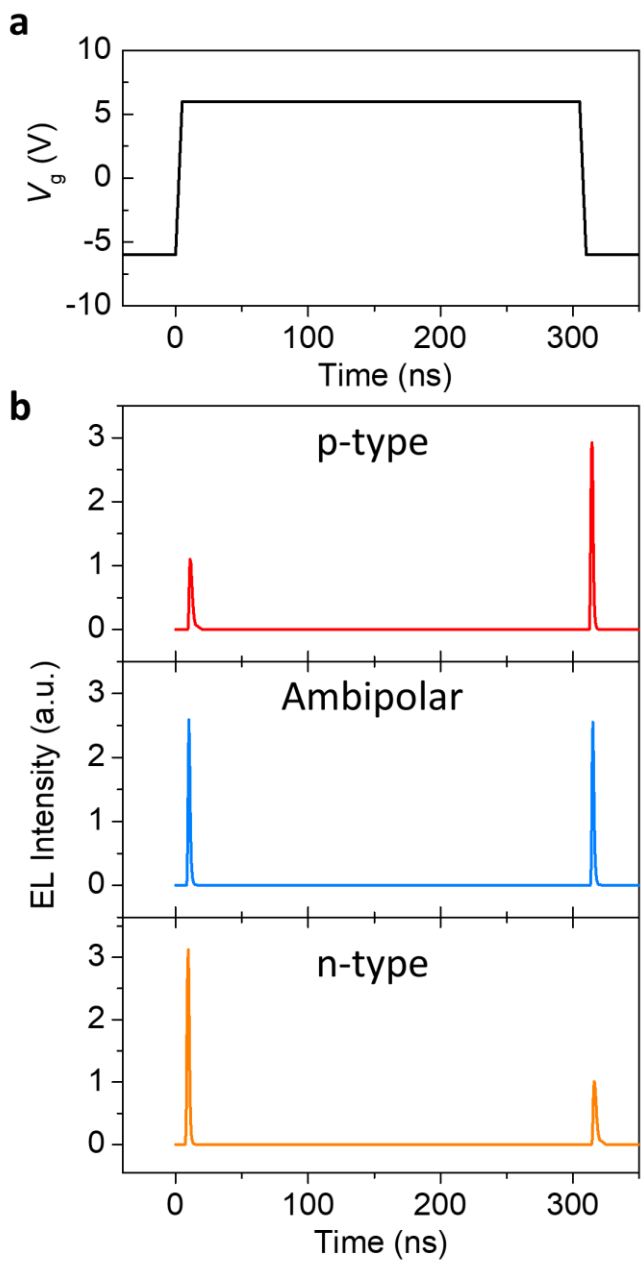

Supplementary Figure 11 | Simulated TREL for p-type, ambipolar and n-type devices. (a) Gate voltage as a function of time. **(b)** Simulated TREL for devices with varying Schottky barrier height; specifically, the cases of Fermi level pinning at conduction band (n-type), mid-gap (ambipolar) and valance band (p-type). Simulations were performed for material parameters corresponding to WSe_2, further details are provided in the methods.

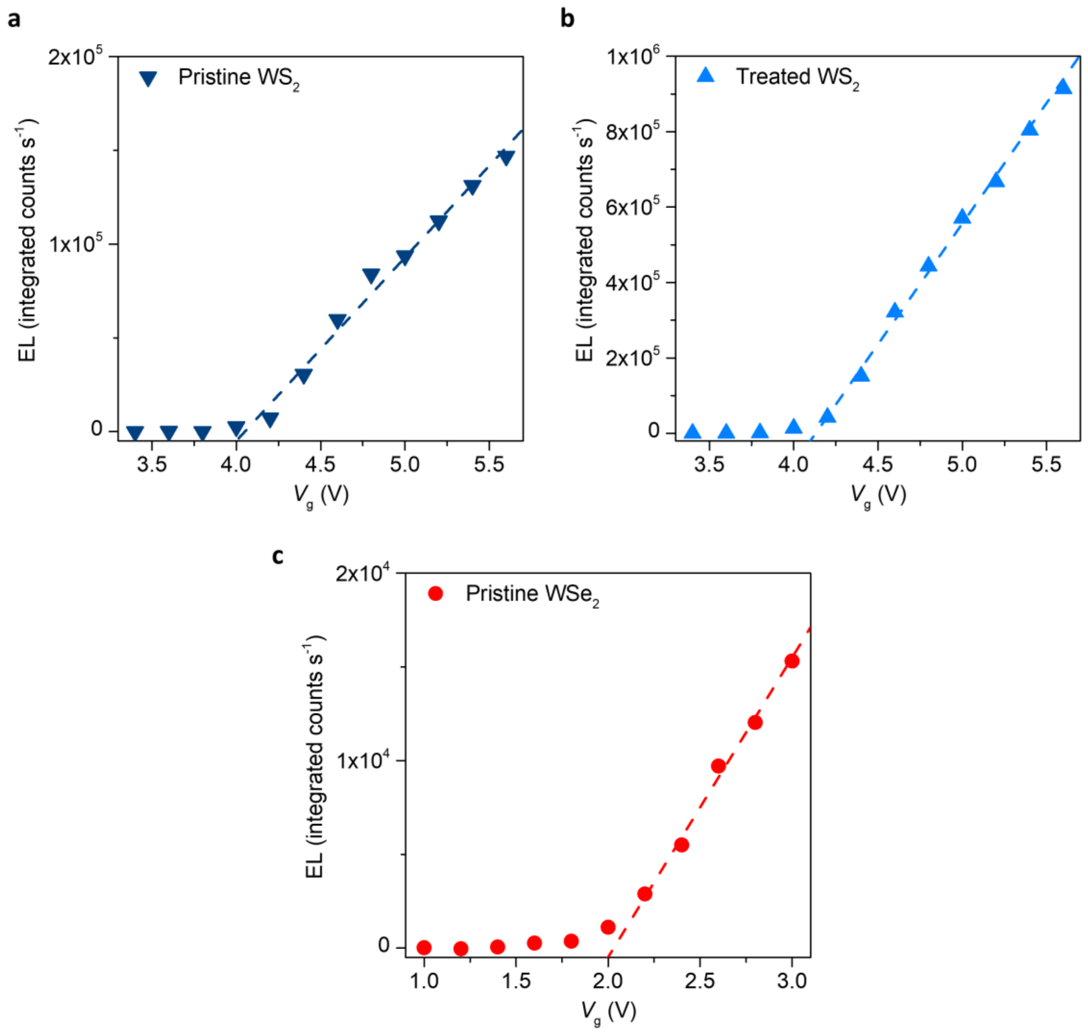

Supplementary Figure 12 | V_g dependence of EL characteristics. EL measured for a WS_2 device before **(a)**, and after superacid treatment **(b)**, as well as a WSe_2 device **(c)**, as a function of injected carrier concentration. Note that V_t for the WSe_2 and WS_2 devices are 2.0 V and 4.1 V, respectively.

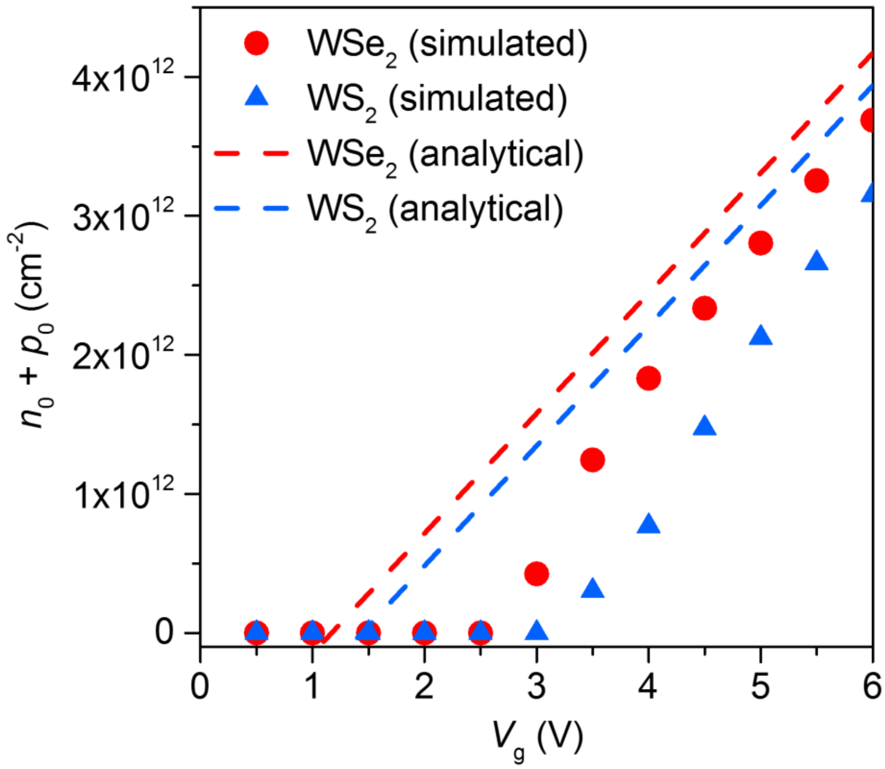

Supplementary Figure 13 | Total steady-state injected carrier concentration as a function of applied voltage. n_0+p_0 extracted from simulations and calculated using Supplementary Equation 4 for WSe_2 and WS_2 devices as a function of applied V_g. The injected carrier concentration at low V_g is overestimated by Supplementary Equation 4 because it does not account for the voltage dropped across the length of the semiconductor during a V_g transient. E_g values used for the simulations and analytical calculations are 2.34 eV and 2.88 eV for WSe_2 and WS_2 respectively[2].

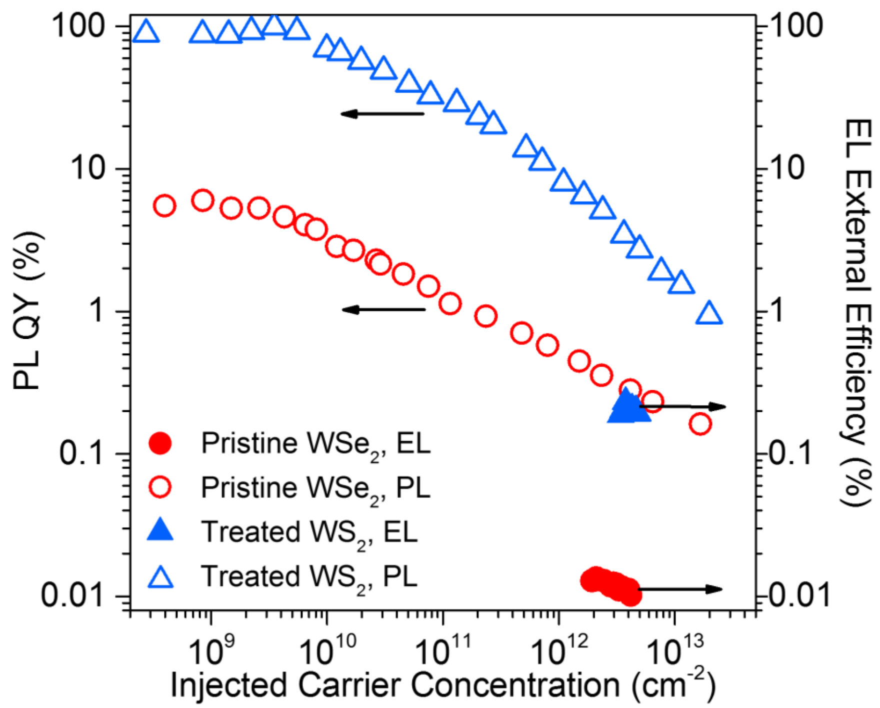

Supplementary Figure 14 | PL QY and EL external efficiency. PL QY and EL external efficiency as a function of injected carrier concentration measured for a WSe_2 device and a superacid-treated WS_2 device.

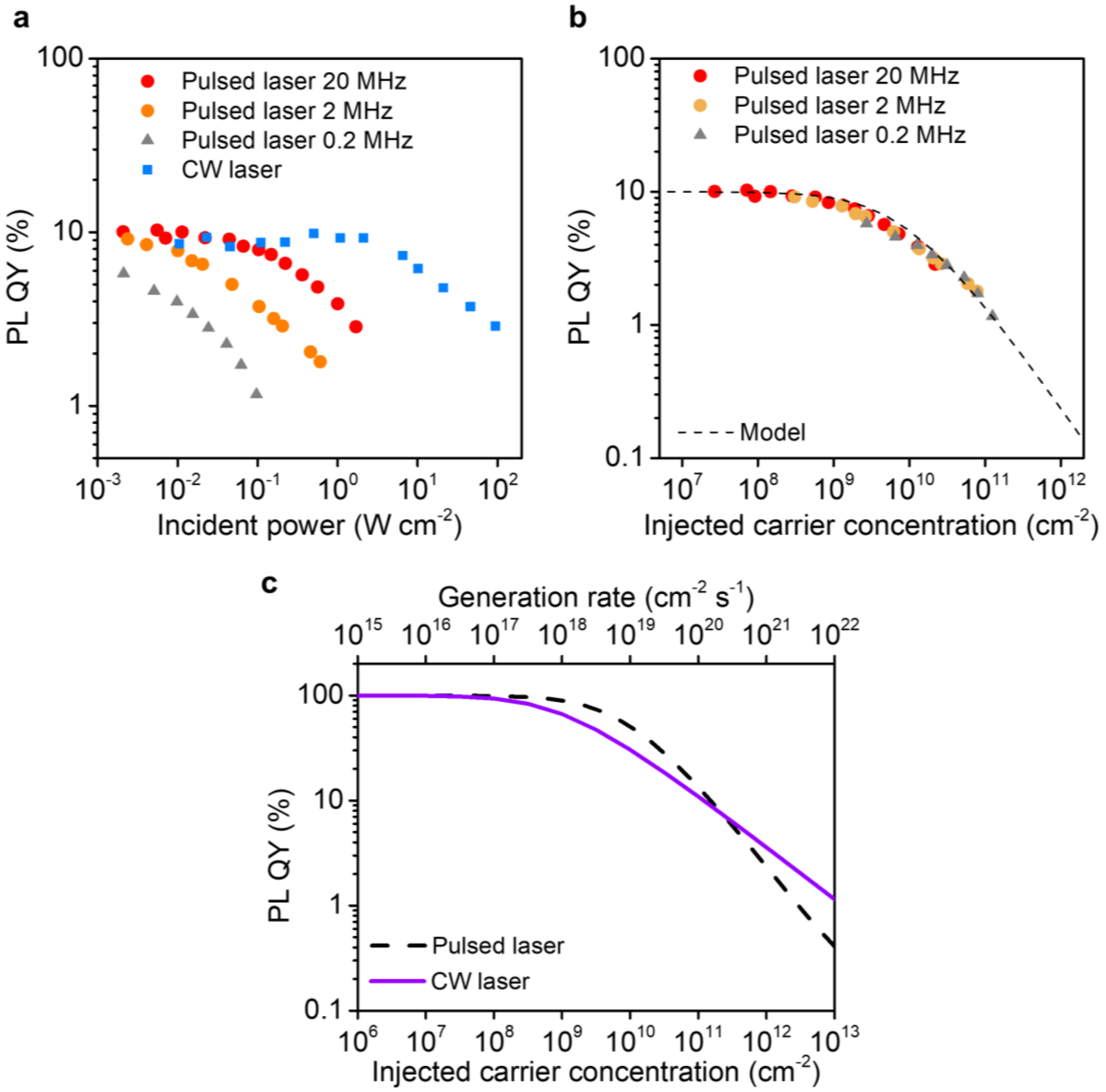

Supplementary Figure 15 | Quasi-steady-state PL QY. (a) QY measured as a function of incident average power for a CW laser and a pulsed laser with repetition rates of 0.2, 2, and 20 MHz; excitation wavelength is 514 nm in all cases. **(b)** Quasi-steady-state QY as a function of incident carrier concentration from measurements shown in panel **(a)**; dashed line shows recombination model. **(c)** Modeled recombination for steady state (purple) and quasi-steady-state (black) excitation.

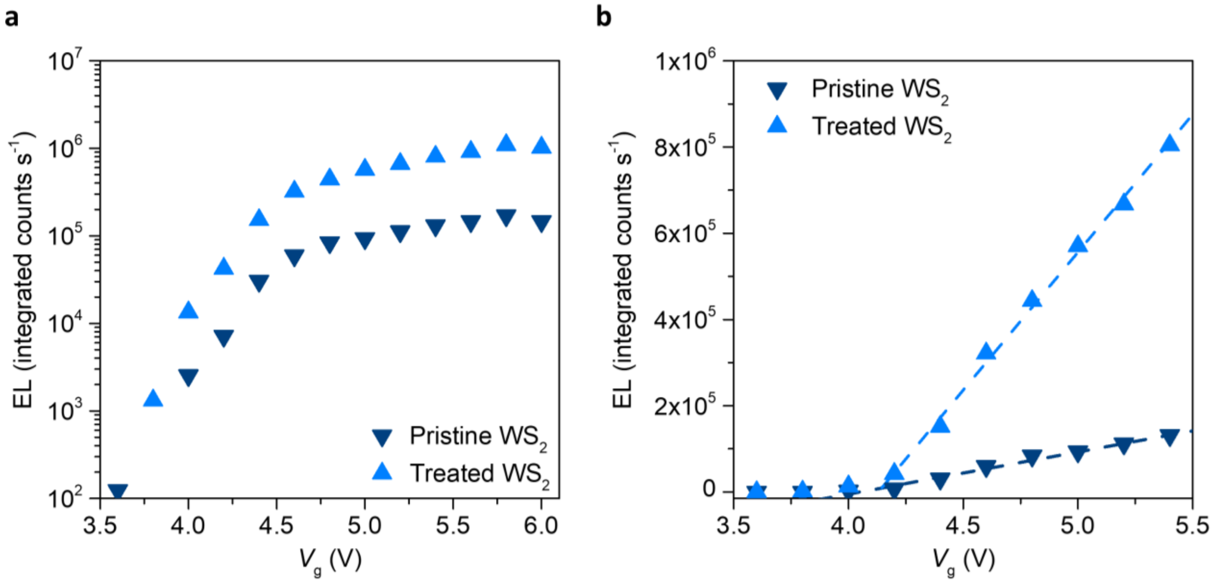

Supplementary Figure 16 | EL from WS_2 before and after superacid treatment. V_g dependence of EL from a WS_2 device before (pristine) and after superacid treatment (treated), plotted in **(a)** logarithmic and **(b)** linear scale.

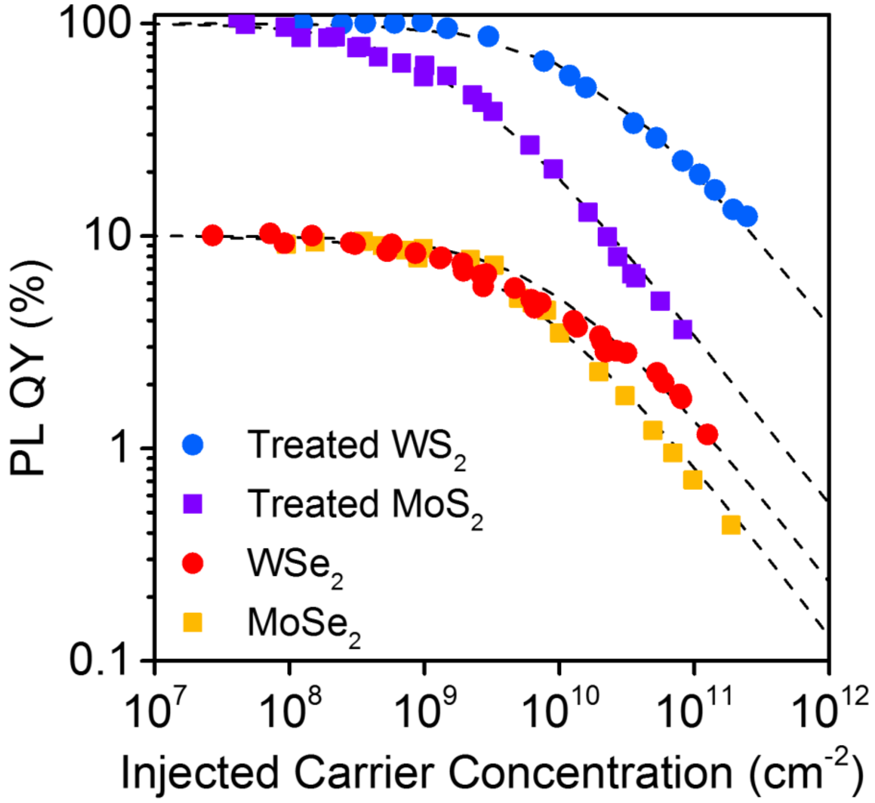

Supplementary Figure 17 | Quasi-steady state PL QY of TMDCs. PL QY of exfoliated $MoSe_2$, CVD WSe_2, and of superacid treated WS_2 and MoS_2 measured using a pulsed laser. Dashed lines show recombination model.

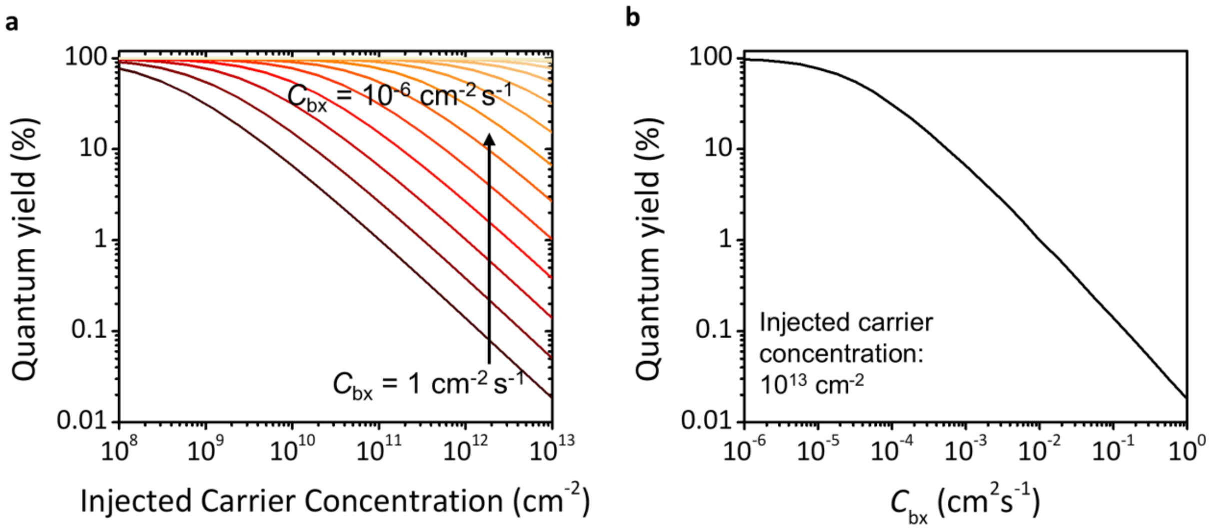

Supplementary Figure 18 | Estimation of PL QY for materials with varying C_{bx}. (a) An estimation of efficiency versus injected carrier concentration while tuning the C_{bx} of a material with a PL radiative lifetime of 10 ns. **(b)** The estimated QY of a material with varied C_{bx} from 1 to 10^{-6} cm^2s^{-1} under the injected carrier concentration of 10^{13} cm^{-2}.

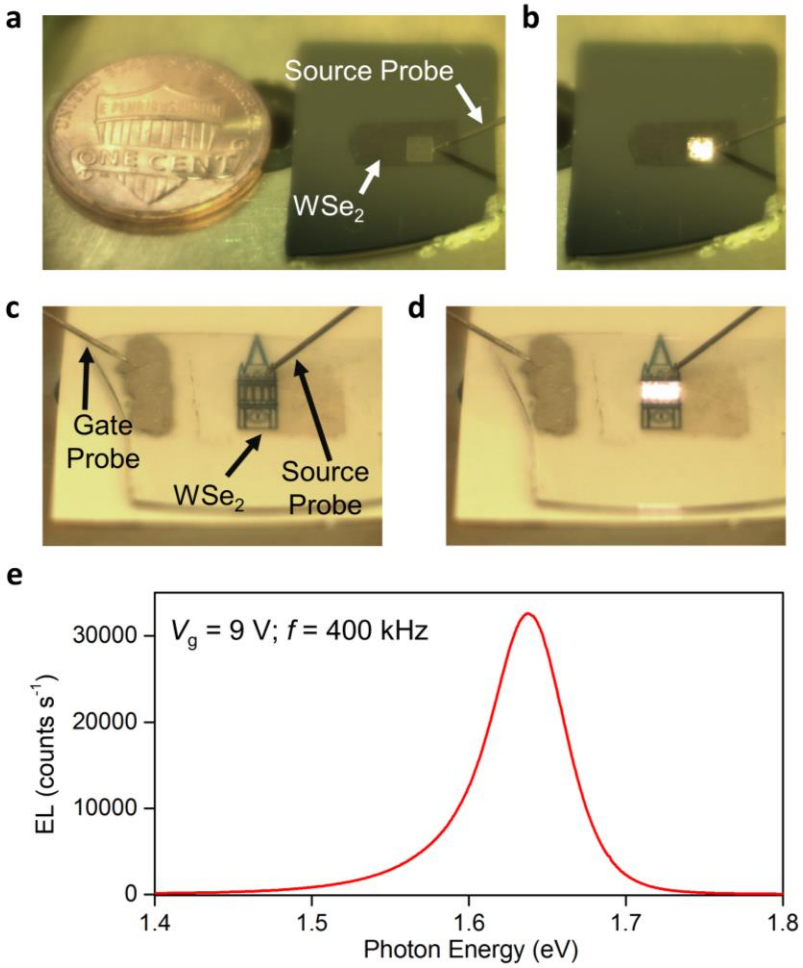

Supplementary Figure 19 | Millimeter-scale t-EL device. (a) Photograph of a WSe_2 t-EL device in the off-state taken in ambient room lights; a penny is shown as a size reference. The device grid structure is schematically shown in Fig. 4d. **(b)** Photograph of the same device in the on-state. **(c)** Photograph of a transparent WSe_2 t-EL device in the off-state, taken in ambient room lights. **(d)** Photograph of the same device in the on-state; note the emission at edges is due to light trapping within the quartz substrate. **(e)** EL spectra of device in panel **(b)** measured with a 10× objective (N.A. of 0.25); note that the device is larger than the objective field of view.

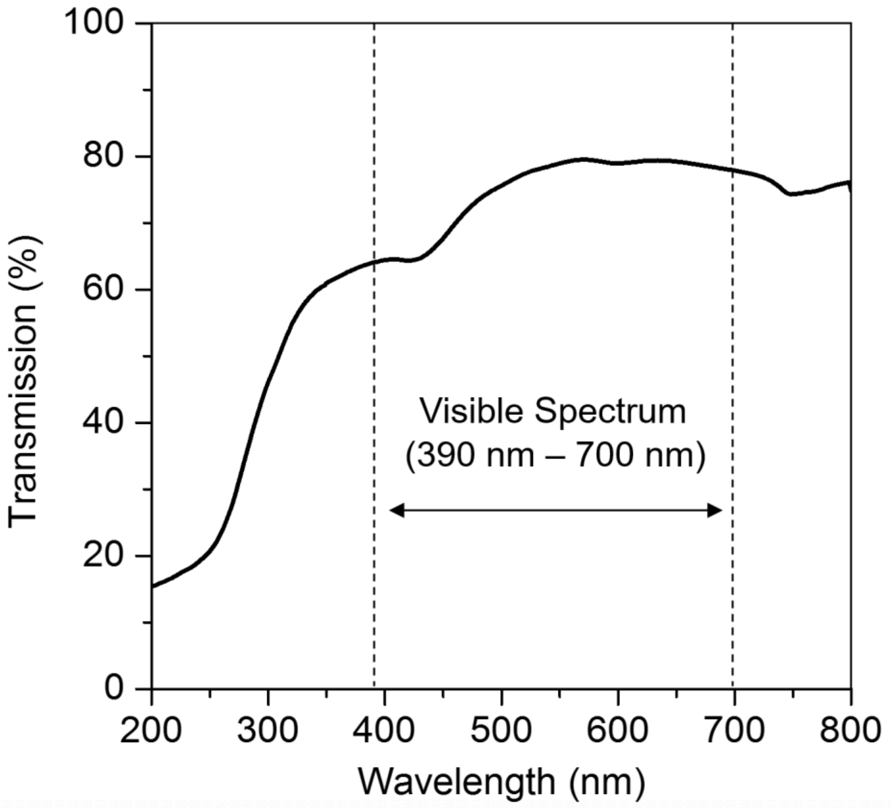

Supplementary Figure 20 | Transmittance of a millimeter-scale t-EL device. Transmission spectrum taken on the millimeter-scale WSe_2 device fabricated on fused quartz, using ITO/Al_2O_3 as the gate stack and ITO as the contact electrode.

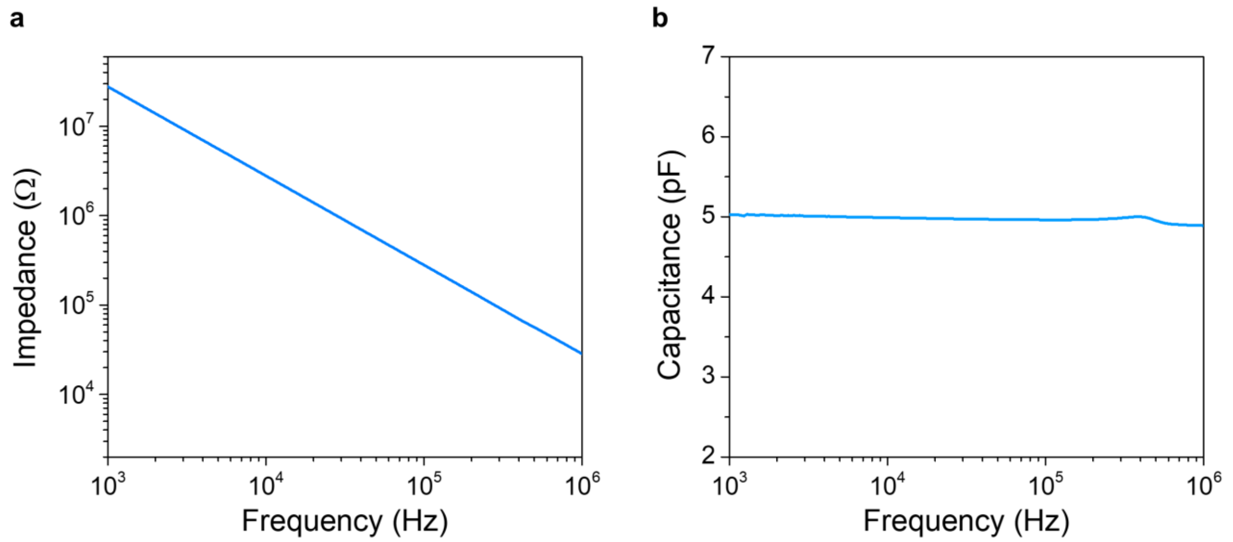

Supplementary Figure 21 | Gate oxide characterization. (a) Measured impedance versus frequency for a WSe_2 device with a 120 μm × 120 μm pad area fabricated on a 50 nm SiO_2/Si substrate. **(b)** Measured capacitance versus frequency for the same device shown in panel **(a)**.

Supplementary Notes

Supplementary Note 1. Quasi-steady state recombination kinetics.

The time dependent luminescence decay in 2D materials can be calculated using a recombination model for 2D excitonic systems presented in our previous work:

$$\frac{d\langle N(t)\rangle}{dt} = -\frac{\langle N(t)\rangle}{\tau_r} - C_{bx}\langle N(t)\rangle^2 \qquad (1)$$

where $\langle N(t)\rangle$ is the exciton concentration as a function of time (t), τ_r is the exciton lifetime, and C_{bx} is the biexcitonic recombination rate[3-5]. Solving this differential equation yields:

$$\langle N(t)\rangle = \frac{\langle N(0)\rangle\, e^{-\frac{t}{\tau_r}}}{1 + \tau_r \langle N(0)\rangle\, C_{bx}\, (1 - e^{-\frac{t}{\tau_r}})} \qquad (2)$$

where the boundary condition, $\langle N(0)\rangle$, is the initial exciton concentration. From Supplementary Equation 2, we can then calculate the PL QY during quasi-steady-state operation as a function of initial exciton density. The modeled values are in excellent agreement with quasi-steady-state QY measured using a pulsed laser with varying repetition rates as shown in Supplementary Fig. 15.

Supplementary Note 2. AC frequency dependence.

EL spectra measured with f ranging from 100 kHz to 10 MHz are shown in Supplementary Fig. 3a and the spectral shape was observed to be f independent (Supplementary Fig. 3b). The EL intensity per cycle shows minimal frequency dependence because $1/f$ is much larger than the EL decay time constant (~ 8 ns, Supplementary Fig. 2). This shows that the device can be operated at frequencies as high as 10 MHz, and is ultimately limited to a modulation frequency of 125 MHz corresponding to the EL decay time constant. This is the fastest electrically modulated light emitting device reported for TMDCs[6].

Supplementary Note 3. Current components during AC transient.

The total gate current ($I_{g,total}$) in a two-terminal t-EL device can be described by Supplementary Equation 3:

$$I_{g,total} = I_{g,DC} + I_{g,AC} \approx I_{g,AC} = I_{g,displacement} \quad (3)$$

where, $I_{g,DC}$ is the DC leakage current and $I_{g,AC}$ is the AC current at the gate electrode induced by the applied AC bias (V_g transients). From steady-state I_d-V_g measurements and capacitance measurements of the gate impedance as a function of frequency, we observe that the gate oxide shows no leakage current. Thus, the total gate current is mainly composed of the AC current, which is the displacement current generated by the changing electric fields in the device during a V_g transient. The corresponding current at the source terminal can be described by Supplementary Equation 4.

$$I_{s,total} = I_{s,DC} + I_{s,AC} \approx I_{s,AC} \quad (4)$$

The DC component of the current is again negligible. The AC current at the source contact is a combination of the electron, hole and displacement currents as shown in Supplementary Equation 5.

$$I_{s,AC} = I_{s,hole} + I_{s,electron} + I_{s,displacement} \quad (5)$$

It is not possible to experimentally measure these components separately, however Sentaurus simulations can help us understand the current components in more detail. Simulations (Supplementary Fig. 7) indicate that the major current components at the source electrode are the electron and hole currents, and their relative magnitude depends on the particular edge in the V_g transient. The simultaneous presence of electrons and holes in the semiconductor leads to EL emission.

Supplementary Note 4. EL dependence on Schottky barrier heights.

For devices with varying Schottky barrier (φ_B) heights, the relative intensity of EL at each V_g transient also shows large variations, although the integrated EL per cycle remains independent of φ_B. This can be qualitatively explained considering the specific example where we have hole selective; contacts to the semiconductor. In this case, during a -V_g to +V_g transient, the level of electrons which tunnel into the semiconductor is very low because of the large Schottky barrier height to electrons. This results in a smaller net bipolar carrier concentration in the semiconductor which translates to lesser EL. However, during the +V_g to -V_g transient, a large number of holes are injected into the semiconductor because of the smaller barrier height, resulting in a net larger bipolar carrier concentration, and hence larger EL. For the case of n-type contacts to the semiconductor, the mechanism can be similarly explained, and we obtain larger EL during the -V_g to +V_g transient relative to the +V_g to -V_g transient.

Supplementary Note 5. Gate oxide characterization.

To verify the gate oxide quality as well as to directly measure C_{ox}, capacitance vs. frequency measurements were performed on devices fabricated on 50 nm SiO_2/Si substrates as shown in Supplementary Fig. 21 over a frequency range of 1 kHz to 1 MHz. The measured capacitance of 5.1 pF is consistent with the calculated value of 6.91 pF. Furthermore, the measured impedance vs. frequency is in excellent agreement with the expected impedance of a direct capacitor (which is given as $X_c = (2\pi f C_{ox})^{-1}$, where X_c is the reactance of the gate capacitor). Furthermore, from DC measurements the leakage currents are below the noise level (pA range) of the measurement setup, and indicate that the device has a purely reactive impedance.

Supplementary Note 6. Efficiency calculation.

The EL internal efficiency of the t-EL device can be extracted from the ratio of the total number of emitted photons per cycle to the steady-state electron (n_0) and hole (p_0) concentrations:

$$\eta_{\mathrm{i}} = \frac{\int_0^T \int_0^L R\,dx\,dt}{(n_{\mathrm{o}} + p_{\mathrm{o}})L} = \beta(\mathrm{PL\ QY}) \tag{6}$$

Here T is the time period, L is the length of the device, R is the radiative recombination rate and β is the fraction of steady-state carriers which undergo recombination during a V_g transient. The value of (n_0+p_0) can be calculated using Supplementary Equation 6. During a -V_g to +V_g transient (total change of 2V_g), the net voltage dropped at the Schottky source contact is equal to the sum of the barrier heights to electrons and holes ($\varphi_{Bn} + \varphi_{Bp} = E_g$). Thus,

$$(n_{\mathrm{o}} + p_{\mathrm{o}}) = \frac{C_{\mathrm{ox}}\left[2V_{\mathrm{g}} - \left(\varphi_{\mathrm{Bn}} + \varphi_{\mathrm{Bp}}\right) q^{-1}\right]}{q} \tag{7}$$

To achieve steady-state carrier densities, sufficient voltage must be applied to enable large band bending and thus achieve significant tunneling through the Schottky barriers. As such this equation is only valid for sufficiently high V_g, which is shown in Supplementary Fig. 13. The integral in the numerator is equal to the total number of photons emitted per cycle. The internal efficiency is unity when the PL QY is 100% and all of the steady-state carriers undergo recombination (β = 1). Practically, due to the finite slew rate of the AC source and a finite radiative recombination rate, a fraction of the steady-state carriers exit the semiconductor through the source contact without recombining ($\beta \neq 1$). The external efficiency is given by:

$$\eta_{\mathrm{e}} = \eta_{\mathrm{i}}\,\eta_{\mathrm{ext}} \tag{8}$$

where η_{ext} is the light extraction efficiency. This is calculated using $(4n^2)^{-1}$ where n is the refractive index of the medium, as well as the optical interference from the Si substrate with a 50 nm SiO_2 layer. The enhancement factor for Si/SiO_2 is experimentally determined to enhance the light-outcoupling by 1.6× for WSe_2 and 2× for WS_2.

Supplementary Methods

Three independent approaches were used to verify the system calibration, and are discussed in detail in our previous work.[3] In the first approach, the wavelength of the spectrometer was calibrated using Ar and Kr lamps (Newport) as reference. The instrument function versus wavelength of the system was obtained by measuring the response of a Lambertian light source generated under the objective through the illumination from a temperature-stabilized lamp (ThorLabs SLS201) onto a diffuse reflector surface. The system efficiency was calibrated by measuring the response of the 514 nm laser focused on the diffuse reflector. In the second approach, we cross-calibrated using a silicon photodiode which was able to acquire a fraction of the PL but is independent of the optical path of objective. In the third approach, the calibrations were performed using a sample with a known QY close to 100% (Rhodamine 6G in methanol). These three approaches were in good agreement with each other (<15% error).

Supplementary References

1. Chuang, S. *et. al.* MoS_2 p-type transistors and diodes enabled by high workfunction MoO_x contacts. *Nano Lett.* **14**, 1337-1342 (2014)
2. Ramasubramaniam, A. Large excitonic effects in monolayers of molybdenum and tungsten dichalcogenides. *Phys. Rev. B* **86**, 115409 (2012).
3. Amani, M. *et. al*. Near-unity photoluminescence quantum yield in MoS_2. *Science* **350**, 1065-1068 (2015).
4. Amani, M. *et. al*. Recombination kinetics and effects of superacid treatment in sulfur- and selenium-based transition metal dichalcogenides. *Nano Lett.* **16**, 2786-2791 (2016).
5. Kim, H., Lien, D.-H., Amani, M., Ager, J. W. & Javey, A. Highly stable near-unity photoluminescence yield in monolayer MoS_2 by fluoropolymer encapsulation and superacid treatment. *ACS Nano* **11**, 5179-5185 (2017).
6. Liu, C.-H. *et. al.* Nanocavity integrated van der Walls heterostructure light-emitting tunneling diode. *Nano Lett.* **17**, 200-205 (2017).
