# Supplementary material for: Large-area and bright pulsed electroluminescence in monolayer semiconductors
Source: Nat Commun. 2018 Mar 26;9:1229. doi: 10.1038/s41467-018-03218-8 (PMC5955902; doi:10.1038/s41467-018-03218-8)
Supplement: Supplementary file 2 — Description for the Supplementary Movies [file 41467_2018_3218_MOESM2_ESM.docx]

Description for the Supplementary Movies

Supplementary Movie 1: An introduction of the t-EL device

Supplementary Movie 2: Operation of a millimeter-scale t-EL device

Supplementary Movie 3: Operation of a transparent t-EL device
